# 凝血功能与非小细胞肺癌相关机制研究进展

**DOI:** 10.3779/j.issn.1009-3419.2013.12.11

**Published:** 2013-12-20

**Affiliations:** 050000 石家庄，河北医科大学第四医院肿瘤内科 Department of Oncology, the Fourth Hospital of Hebei Medical University, Shijiazhuang 050000, China

**Keywords:** 凝血功能, 肺肿瘤, 病理生理学, Blood coagulation, Lung neoplasms, Physiopathology

## Abstract

近来研究越来越多地发现凝血功能紊乱通常是恶性肿瘤的首发迹象。现在已经证实，肿瘤导致血栓形成的风险增加，而凝血功能的过度激活也极大地影响肿瘤的进展。在肺癌患者中，存在着持续的凝血刺激。癌细胞通过组织因子（tissue factor, TF）的表达激活凝血功能；通过凝血酶的表达和促凝血微粒的释放等影响凝血功能。凝血功能也通过介导血小板释放其颗粒内容物、抑制自然杀伤细胞和募集巨噬细胞而促进肿瘤的进展。非小细胞肺癌（non-small cell lung cancer, NSCLC）占肺癌的80%-85%，本文就凝血系统各个组分在NSCLC发生发展中的病理生理学机制的最新研究进展进行综述。

## Abstract (en)

Recently, researchers have been increasingly finding coagulation disorders are commonly the first sign of malignancy. It has now been established that cancer development leads to an increased risk of thrombosis, and conversely, excessive activation of blood coagulation profoundly influences cancer progression. In patients with lung cancer, a sustained stimulation of blood coagulation takes place. Cancer cells trigger coagulation through expression of tissue factor, and affect coagulation through expression of thrombin, release of microparticles that augment coagulation and so on. Coagulation also facilitates tumour progression through release of platelet granule contents, inhibition of natural killer cells and recruitment of macrophages. Non-small cell lung cancer (NSCLC) accounts for about 80%-85% of all lung malignancies. In the present review, we summarized the newly updated data about the physiopathological mechanisms of various components of the clotting system in different stages of carcinogenesis in NSCLC.

在过去的30年中，相关研究已经证实凝血系统极大地促进了肿瘤的进展和转移。50%的癌症患者存在凝血功能的异常，转移性病变中凝血功能异常的概率高达90%^[1]^。凝血系统参与细胞从休眠状态、非血管化肿瘤转化到高转移表型的多次相互作用。肿瘤将凝血系统活化到较高水平，则其具有更强的侵袭性生物学行为。Armand Trousseau教授于1865年首先报道了恶性肿瘤与血栓形成的相关性，肿瘤患者体内往往呈高凝状态，有并发血栓性疾病的潜在风险。静脉血栓栓塞症（venous thrombo embolism, VTE）已成为导致肿瘤患者死亡的第2位原因，其中深静脉血栓栓塞（deep venous thrombosis, DVT）与肺血栓栓塞（pulmonary thrombo-embolism, PTE）是实体恶性肿瘤最常见的并发症^[2]^，并且在肺癌患者中发病率较高^[3]^。肺癌已是世界上发病率和死亡率最高的恶性肿瘤之一^[4]^，不同病理类型及分期的肺癌其VTE的发生率不同，腺癌是鳞癌的2倍-3倍，非小细胞肺癌（non-small cell lung cancer, NSCLC）是小细胞肺癌的1.5倍-2.1倍，而远处转移的患者较局限病灶者高4倍-6倍^[5]^。肺癌通过多种复杂机制破坏机体内凝血和抗凝血之间的动态平衡，从而导致机体的凝血功能紊乱，这不仅与肿瘤的生长、浸润、侵袭、转移以及并发血栓性疾病等密切相关，而且还直接影响肺癌患者的预后。凝血功能与肺癌密切相关，本文就凝血系统各个组分在NSCLC发生发展中的病理生理学机制的最新研究进展进行综述。

## 1 组织因子（tissue factor, TF）

TF是位于细胞表面相对分子量为47 kDa的膜蛋白，是凝血级联反应过程中主要的激活因子。正常的内皮细胞不表达TF，而在组织损伤、炎症或肿瘤等病理情况下，肿瘤坏死因子-α、白介素-1β等可诱导血管内皮细胞、肿瘤细胞或单核/巨噬细胞大量表达TF。TF与FⅦ结合，形成TF-FⅦa复合物，从而激活FX、FIX，启动外源性凝血途径。TF负责肿瘤微环境中局部凝血酶的形成和纤维蛋白的沉积，从而影响肿瘤和宿主细胞之间多种细胞相互作用。

癌细胞是一个促凝血刺激因素，其促凝机制包括细胞表面TF的表达^[6]^。肿瘤特定类型的致癌转化导致癌细胞TF的上调，提高肿瘤微环境的促凝特性，促进肿瘤的进展、血管生成和转移^[7]^。TF可以通过蛋白酶活化受体（protease-activated receptors, PARs）家族介导信号的转导，PARs分为PAR-1、PAR-2、PAR-3和PAR-4四种亚型。TF-FⅦa复合物激活肿瘤细胞表面PAR-2^[8]^或整合素，通过MAPK及JNK信号转导通路上调血管内皮生长因子（vascular endothelial growth factor, VEGF）表达，同时抑制抗血管生成蛋白如凝血酶敏感蛋白-1（thrombospondin-1, TSP-1）的表达^[9]^。TF通常是粘附到细胞表面，但它也可以定位于血液中由血管内细胞如血小板、血管内皮细胞、白细胞以及肿瘤细胞表面脱落的亚微米泡的表面。这些囊泡被称为微粒（microparticles, MPs），多项研究发现，血浆中MP-TF的浓度或MP-TF的促凝活性与VTE的风险甚至与VTE的复发呈正相关。另一研究^[10]^发现MP-TF与癌症患者的死亡率相关，而与其血栓形成无明显关联。TF和整合素β1都定位于MPs，MP与整合素β1的连接可以作为一种粘附分子，促进MP持续地结合于细胞外基质（extracellular matrices, ECMs）如胶原蛋白和纤连蛋白，因此促进MP-TF的凝血活性^[11]^。TF也可以通过一个称为选择性剪接的TF（alternatively spliced TF, asTF）的剪接异构体促进肿瘤的血管生成。asTF可以调节内皮细胞的功能，并通过上调粘附受体来诱导白细胞的募集^[12, 13]^。asTF与整合素α6β1和αvβ3结合，调节内皮细胞的迁移并促进体内肿瘤的活动^[11]^。TF的过表达是肿瘤相关血栓形成的一个重要因素，并促进原发肿瘤的生长^[14]^。研究^[15]^表明，在肺癌转移的早期阶段，癌栓的形成对于癌细胞的生存是必要的。

研究^[16]^发现NSCLC中TF的表达是升高的，而且与肿瘤的分期相关。对NSCLC原发肿瘤的分析表明，TF抗原与VEGF的表达和血管生成直接相关；TF的表达与生存时间呈负相关^[17]^。在一组NSCLC细胞系中TF的下调减少了裸鼠试验肿瘤的生长^[18]^。TF、基质金属蛋白酶（matrix metalloproteinase, MMP）的表达以及粘附在癌症转移中是高度相关的，研究^[19]^表明TF表达小细胞肺癌H69细胞的可逆性生长表型。

## 2 凝血酶

凝血酶原（prothrombin)（又称FII）释放凝血酶原片段1+2（prothrombin fragment 1+2, F1+2）产生凝血酶。凝血酶是一种功能强大的蛋白水解酶和促凝化合物，能够增强肿瘤细胞对细胞因子的增殖反应，增强肿瘤细胞对血小板（PLT）的黏附及细胞基质的侵袭，促进肿瘤血管形成和肿瘤微环境的组织重建。肺癌F1+2水平升高，VTE发生的危险性增加^[20]^。在小鼠模型中，凝血酶原和纤维蛋白原的不足、小鼠血小板的损伤、血小板凝血酶受体PAR4缺失和内皮凝血酶敏感蛋白^[21]^对凝血酶的调节，直接或间接抑制凝血酶，从而降低了肿瘤细胞的转移潜能。

凝血酶通过PARs介导信号转导，通过MAPK、ERK和PI3-K信号通路，上调VEGF、MMP-2、IL-8和整合素等的表达，促进肿瘤的生长、浸润及转移^[22]^。PARs中PAR-1和PAR-2是主要的凝血酶受体，在肿瘤组织及微环境中表达增高。凝血酶和PAR-1结合，上调PLT衍生生长因子、PLT激活因子及其受体的表达，激活PLT，促进肿瘤的生长和转移^[23]^。凝血酶激活MMP-2，破坏基底膜，促使内皮细胞在新的肿瘤纤维蛋白基质中增殖。

## 3 纤溶系统和抗凝系统

### 3.1 纤溶系统

在肿瘤生长、血管生成和凝血级联反应激活过程中，纤溶系统也被激活。这个过程涉及纤维蛋白的溶解和防止有缺陷的肿瘤血管中的血栓形成。纤溶系统包括血纤维蛋白溶酶酶原（plasminogen, PLG），它可转化为有活性的血纤维蛋白溶酶。纤溶酶负责纤维蛋白的降解，也可活化MMPs，从而降解细胞外基质。D-二聚体是交联的纤维蛋白在纤溶酶作用下裂解产生的一种特异性的代谢产物，其存在能够表明体内有纤维蛋白形成和溶解，即血栓形成和溶解。D-二聚体是纤溶过程的一个敏感的标记物，高D-二聚体浓度水平的肺癌患者较低水平者预后差^[24]^。

纤溶酶原可由两个不同的纤溶酶原激活剂（组织型纤溶酶原激活剂（tissue type plasminogen acitivator, tPA）和尿激酶型纤溶酶原激活剂（urokinase type plasminogen activator, uPA）活化为纤溶酶。t-PA介导的纤溶酶原的激活主要参与循环中纤维蛋白的溶解；而u-PA介导的纤溶酶原的活化主要与细胞外蛋白的水解相关，如组织重构和肿瘤的侵袭^[25]^。纤溶酶原激活系统（尤其是u-PA）在肿瘤的侵袭和转移中起重要作用。u-PA/u-PAR系统通过增加细胞外基质的降解和局部蛋白的水解，促进肿瘤的侵袭；同时可通过促血管新生作用和u-PA/u-PAR信号通路使肿瘤向更加恶性的表型转化。uPA高表达或与内皮素-1（endothelin-1, ET-1）同时高表达的肺腺癌患者具有较长的术后生存时间^[26]^。uPAR由三个同源结构域Ⅰ、Ⅱ、Ⅲ组成。研究^[27]^表明，血清中uPAR（Ⅰ-Ⅲ）和uPAR（Ⅰ）是根治性手术NSCLC患者的独立预后因素。

纤溶酶原/纤溶酶系统激活后，通过α2-抗纤溶酶调节纤溶酶的水平^[28]^，通过纤溶酶原激活剂抑制剂（如PAI-1、PAI-2、PAI-3）或间接通过凝血酶激活纤溶抑制剂（thrombin-activatable fibrinolysis inhibitor, TAFI）控制纤溶酶原的活化水平。PAI-1的抗纤溶作用有助于循环肿瘤细胞与内皮细胞的粘附，并启动肿瘤细胞外渗及转移灶形成的过程。PAI-1通过上调c-jun/ERK、Bcl-2、Bcl-XL和下调Bcl-X8、Bax，促进肿瘤细胞的存活^[29]^。PAI-1还通过抑制FAS/FASL信号通路调节细胞凋亡，并促进血管生成^[30]^。研究表明，在肺成纤维细胞中，PAI-1可通过增加细胞内的Ca^2+^浓度及促进细胞周期的进行，从而激活ERK和AKT信号通路^[31]^。99例肺腺癌患者的uPA和PAI-1对总生存预后影响的研究表明，PAI-1高于平均水平则预后差；低水平PAI-1、高水平uPA-PAI-1复合物的患者比高水平PAI-1、低水平uPA-PAI-1复合物者有更好的生存^[32]^。

### 3.2 抗凝系统

抗凝血酶Ⅲ（ATⅢ）和维生素K依赖性凝血抑制剂（蛋白C和S）与增强的促凝血活性相抗衡。ATⅢ是最重要的天然凝血酶抑制剂。活化的蛋白C（APC）使FVa和FVⅢa失活，蛋白S（PS）作为其辅助因子发挥抗凝作用。APC可以与受体PAR-1或EGFR结合，启动MAPK和PI3K信号途径促肿瘤的生长，与细胞外MMP-2和MMP-9作用降解ECM，从而促进肿瘤转移^[33]^。

肺癌转移是基于复杂的相互作用，触发肿瘤及其周边环境的信号通路，调节肿瘤细胞的存活及器官定植^[34]^。血管内皮细胞蛋白C受体（endothelial cell protein C receptor, EPCR）是高度表达于血管内皮的跨膜受体，其主要的天然配体是蛋白C和APC。APC与EPCR结合后增强抗氧化、抗炎及抗血栓形成的功能。肺腺癌是肺癌最常见的组织学亚型，转移是其常见的并发症^[35]^。研究发现I期肺腺癌患者的EPCR水平与其生存期有明显的相关性；高水平的EPCR与无疾病进展生存（progression free survival, PFS）明显相关，这提示EPCR是肺腺癌疾病进展的独立危险因素；肿瘤细胞表达EPCR，APC与EPCR结合，促进肿瘤细胞的存活，提高了肺腺癌的转移活性，并抑制肺腺癌细胞的凋亡，有助于肺癌的转移^[36]^。

## 4 血细胞和细胞因子

### 4.1 血小板

研究^[37]^发现30%-60%的肿瘤患者有血小板增多，血小板增多在肺癌患者中较普遍。肿瘤细胞所致的血小板聚集高度支持肿瘤转移^[38]^，血小板与肿瘤细胞的相互作用是肿瘤成功转移必不可少的一部分。血小板通过释放其颗粒中的各种生长因子和趋化因子（如PDGF、IGF-1、VEGF等），上调其它促血管新生介质（包括基质细胞衍生因子CXCL12、MMP-1、MMP-2和MMP-9）的表达，刺激肿瘤的生长，影响血管生成^[39, 40]^。最近研究^[41]^表明，血小板在肿瘤转移的早期阶段也发挥重要的作用，活化的血小板释放大量转化生长因子β（TGFβ），激活TGFβ/Smad和NF-κB信号转导通路，诱导肿瘤细胞类上皮间质转化（epithelial-mesenchymal transition, EMT），提高肿瘤细胞的转移潜能。TGFβ是一个细胞因子超家族成员，能够调节一系列细胞功能，在肺疾病（包括肺癌）中起重要作用。TGFβ1通过MEK1/ERK和PI3K/Akt信号通路介导肺癌的转移^[42]^。血小板衍生微粒（platelet derived membrane microparticle, PMP）可表达和转运功能性受体，刺激细胞因子的释放，激活细胞内PI3K-Akt、ERK信号转导通路，促进肿瘤血管生成和转移^[43]^。Gil-Bernabe等^[44]^研究指出TF诱导的血小板聚集与巨噬细胞的募集直接相关，血小板血栓快速募集单核巨噬细胞是肺转移成功的关键。

### 4.2 单核/巨噬细胞

血浆蛋白通过高渗透性的肿瘤血管漏出，可以激活炎症反应和肿瘤诱导的外源性凝血途径。炎症刺激可诱导肿瘤血管内皮细胞的促凝行为。单核/巨噬细胞和T细胞等受到刺激后产生某些细胞因子如IL-1β、TNFα，上调内皮细胞表达TF或PAI-1，促进肿瘤的进展。在NSCLC中IL-6的血清水平与肿瘤的进展和生存相关^[45]^。TF激活凝血系统，促进肿瘤细胞血凝块的形成，引发单核/巨噬细胞的聚集，从而促进肿瘤细胞的存活，这是引发肿瘤转移的一个组成部分^[15]^。研究^[46]^表明，肿瘤有效的转移必须有单核/巨噬细胞的参与，巨噬细胞不仅具有抗肿瘤活性，而且也可以促进肿瘤的发生和进展。

### 4.3 VEGF

血管生成、凝血反应和炎症是与癌症的发生和发展高度相关的复杂的过程。内源性血管生成抑制剂TSP-1和内皮抑素维持生理性的血管生成平衡。内皮抑素是由血小板释放的，可抑制内皮细胞的迁移，诱导细胞凋亡，从而减少肿瘤的血管生成。TSP-1通过调节VEGF的分泌，限制血管生成过程。癌症患者VEGF的上调和TSP-1的下调，导致血管生成控制失调，促进了肿瘤的血管生成和炎症反应。肿瘤缺氧微环境中VEGF表达上调，通过增加VEGF-A的生成诱导血管生成。在血管内皮细胞，VEGF通过其受体VEGFR-2活化MMP，从而激活uPA，导致蛋白水解及肿瘤细胞浸润^[47]^。此外，VEGF也上调内皮细胞uPA、uPAR的表达。在NSCLC患者中，TSP-1的mRNA高表达与较长的PFS相关^[48]^。NSCLC患者的VEGF、其可溶性受体1（VEGFR1）和2（VEGFR2）、IL-6、TF等血管生成、炎症和凝血标志物水平是升高的，高水平的VEGF和低水平的TSP-1与肺癌预后差相关^[49]^。

## 5 展望

在快速增长的肺癌人群中，凝血功能紊乱普遍存在，部分患者出现高血凝状态并发VTE，严重影响患者预后。我们已经掌握了凝血级联反应、纤溶及抗凝系统、血细胞、细胞因子等在肺癌进展和转移中的许多功能和作用机制，然而肿瘤细胞与凝血系统相关的新的分子机制有待进一步的研究，这将有助于针对肺癌和相关凝血功能紊乱进行预防、早期诊断和适当的治疗，可能会对易感人群的临床预后产生明显的影响。
